# Recent Progress of High Performance Thermosets Based on Norbornene Functional Benzoxazine Resins

**DOI:** 10.3390/polym13091417

**Published:** 2021-04-27

**Authors:** Yin Lu, Xinye Yu, Lu Han, Kan Zhang

**Affiliations:** 1 Research School of Polymeric Materials, School of Materials Science and Engineering, Jiangsu University, Zhenjiang 212013, China; a897919092@163.com (Y.L.); yuxinye20@mails.ucas.ac.cn (X.Y.); 2 Oak Ridge National Laboratory, Chemical Sciences Division, Oak Ridge, TN 37831, USA

**Keywords:** benzoxazine, norbornene, polybenzoxazine, high performance

## Abstract

With the growing demand for high performance polymeric materials in industry, several types of thermosets such as bismaleimides, advanced epoxy resins, cyanate esters, and phenolic resins have been widely investigated to improve the performance of thermosetting products. Among them, benzoxazine resins have received wide attention due to their extraordinarily rich molecular design flexibility, which can customize our needs and adapt increasing requirements. To further improve the properties of polybenzoxiazines, researchers have found that the introduction of a norbornene functional group into the benzoxazine moiety can effectively improve the comprehensive performance of polybenzoxazine thermosets. This article focused on reviewing the recent development of high-performance thermosets based on norbornene functional benzoxazine thermosetting resins.

## 1. Introduction

In recent years, the application of high-performance thermosets in the field of aerospace, electronic packing, automotive, and other high-performance areas has been extensively broadened. For example, polyimide, a type of special engineering plastics, is used in separation membrane, microelectronics, and other fields due to its excellent high thermal stability, excellent mechanical properties, and dielectric properties [1]. However, the development of industries and commerce puts forward higher expectations for the performance of materials, which allows researchers to improve the material properties from the perspective of molecular structure. As for benzoxazine resin, adding functionalities or incorporating benzoxazine reactive groups has been successfully applied to improve various properties [2,3,4,5,6].

Polybenzoxazine is a type of high-performance thermosetting resin with outstanding features such as non-shrinkage during the polymerization process [7], high glass transition temperature [8], good thermal and mechanical properties [9,10,11,12,13], low dielectric constant [14,15], and low surface energy [16]. It can be obtained through the thermally activated ring opening of the oxazine ring containing nitrogen and oxygen of benzoxazine monomers in the absence of an initiator or a catalyst [17]. Additionally, the benzoxazine monomers are also easy to synthesize in one step with phenols, amines, and formaldehyde through Mannich condensation [18,19,20,21,22,23]. However, the high temperature required for finishing polymerization, the brittleness of polymerized thermosets, and other shortcomings should also be overcome for further development [24,25].

PMR-15 is one of the most attractive polyimide of the polymerization of monomer reactant (PMR) family designed by the NASA Lewis Research Center [26,27]. Its structure contains a very stable crosslinking network by the polymerization of norbornene end-caps, which gives it excellent thermomechanical properties [28,29]. Due to the outstanding properties in high-temperature performance, it has been widely used in the aerospace industry as composites and adhesives. However, the vulnerability of the cross-links formed from norbornene end-caps under high temperature has seriously impeded its further applications [28].

Based on the above findings and investigations, researchers have turned to focus on introducing norbornene functional groups into benzoxazine to improve the properties of the material and obtain high-performance thermosets or use them as structural units to connect with other monomers to obtain thermosets with better performance. For example, using norbornene to participate in copolymerization to increase the degree of polymerization and obtain a denser network structure, or using the catalytic reaction of norbornene double bonds to form block copolymers to obtain products with specific properties [30,31,32]. These studies broaden the types and applications of norbornene functionalized benzoxazines, and provide great reference value for future related research. Our current review article summarizes the development of high performance thermosets based on norbornene functional benzoxazine resins. Single substituted, disubstituted, *ortho*, *meta*, *para* substituted, mainchain, and side chain type norbornene benzoxazine and siloxane molecules containing norbornene benzoxazines are summarized and the thermal, film forming, molecular weight properties are systematically discussed.

## 2. Norbornene Based Benzoxazine Monomers

**Scheme 1:**
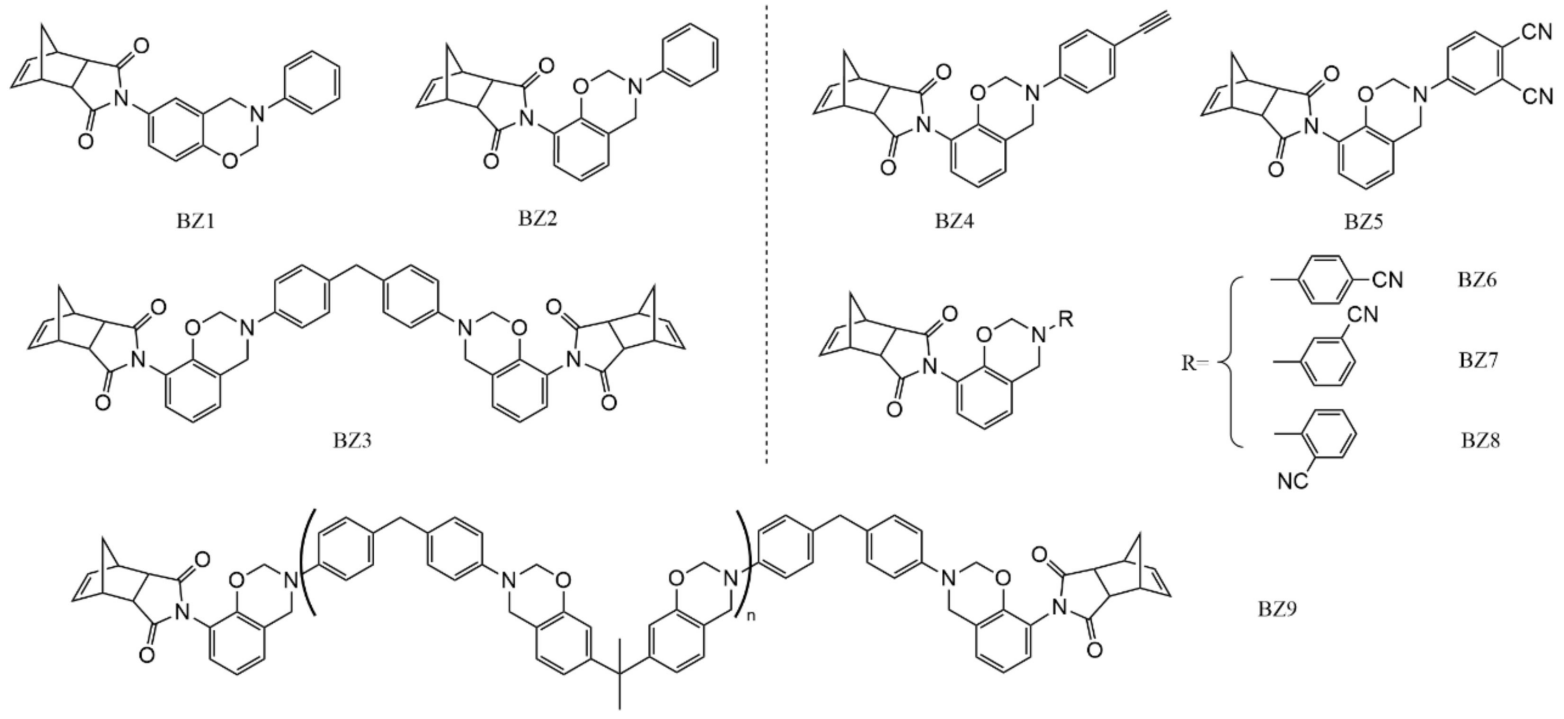
Chemical structures of norbornene based benzoxazines.

As early as 1989, Lyle et al. successfully synthesized maleimide functionalized phenol through the reaction of maleic anhydride and aminophenol under certain conditions, which guided the synthesis of the norbornene functionalized phenol [33]. Based on this work, Ishida and Ohba successfully prepared norbornene functionalized phenol and reacted with formaldehyde and aniline to obtain norbornene based benzoxazine (Scheme 1 BZ1) for the first time in 2005 [30]. They compared the thermal characteristics of norbornene functionalized benzoxazines with other common benzoxazines. Both thermogravimetric analysis (TGA) and differential scanning calorimetry (DSC) showed an increase of char yield above 55% and glass transition temperature *T*_g_ above 250 °C without significantly increasing the viscosity of the monomer. Moreover, the multiple polymerization of vinyl initiated by free radical initiators at different temperatures was also observed, which affected the final polymeric structure of polybenzoxazines. Due to the advantages of norbornene functionality, more studies related to polybenzoxazine fields have begun to focus on norbornene based benzoxazines.

In general, benzoxazines can be divided into *ortho*- and *para*-isomers according to the isomerism of the phenol functional group, and different structures will inevitably lead to different properties of the resulting polybenzoxazines. Ishida et al. studied the polybenzoxazines derived from benzoxazines consisting of *ortho*- and *para*-functional phenolic components [34,35]. Paradoxically, the former monomers were shown to possess significant advantages over the latter ones, which was the opposite to the trend [34,35]. Based on this research, the group developed various *ortho*-norbornene based benzoxazines as shown in Scheme 1. BZ2 and BZ3 used the *ortho*-norbornene functional phenol as the phenolic resource, and aniline and 4,4‘-diaminodiphenylmethane (DDM) as the amino resources, respectively. Whether in terms of synthesis or the properties of the corresponding thermosets, the *ortho*-norbornene functional benzoxazine resins showed excellent features, which contributed to the multiple polymerization based on C=C groups in norbornene groups and oxazine rings. The activation energy of polymerization for two benzoxazines were estimated to be about 90 kJ/mol and 164 kJ/mol. Coexistence of polybenzoxazine networks eliminated the decomposing from the reverse Diels-Alder reaction. In addition, the difunctional polybenzoxazine exhibited excellent thermal performance with the *T*_g_ of 365 °C, and a Yc (char yield) value of 61% [31].

In recent years, our group has been devoted to the synthesis and performance research of *ortho*-norbornene benzoxazine, and various benzoxazines have been successfully synthesized, as shown in Scheme 1 BZ4-BZ9. Containing both norbornene and acetylene functionalities, BZ4 resins showed catalyst-free and low-temperature terpolymerization, which comes from the self-catalytic curing mechanism, possibly attributed to the interaction of the oxazine ring, acetylene group, and norbornene functionality. Because of the terpolymerization of the system and the reduction of free volume caused by it, the coefficient of thermal expansion of the thermoset derived from benzoxazine exhibited a very low value (19.6 ppm/°C), which was much lower than other reported polybenzoxazine thermosets, polyimides, and fully aromatic thermosets based on sydnonealkyne cycloaddition. High thermal stability (T_d5_ ≈ 430 °C) and high char yield value (64%) were also confirmed by TGA. Furthermore, the highly cross-linked polybenzoxazine also showed an exceptionally low dielectric constant (2.98–2.82, in 10 Hz–1 MHz) [36].

Similar to BZ4, BZ5 is synthesized with an *ortho*-norbornene functional phenol and phthalonitrile. It also performs multiple polymerization and self-catalytic polymerization behaviors. What is different is that BZ5 exhibits higher thermal stability with triazine and phthalocyanine structures from the curing of phthalonitrile. It is worth noting that due to the introduction of an *ortho*-norbornene functional group, it is difficult for the cyano functional group to fully participate in the polymerization as steric hindrance during the polymerization process is further consumed, which greatly increases the degree of polymerization and accelerates the process of polymerization. A highly crosslinked system provides the thermoset with high thermal stability and outstanding flame retardancy. The TGA of the resulting thermoset possessed high thermal stability with the *T*_g_ of 419 °C and T_d5_ temperatures of 406 °C (in N_2_) and 412 °C (in air). Moreover, the polybenzoxazine also showed much lower heat release capability than most reported polybenzoxazine thermosts [37].

The group also synthesized BZ6–BZ8 and investigated the isomeric effect of nitrile functionality on the polymerization and thermal properties of *ortho*-norbornene based benzoxazines. As a result, the nitrile functionality was found to be much easier to activate as it is attached at the *ortho*-position in the monomeric structure. Besides, the cross-linked networks formed by the polymerization of the *ortho*-benzoxazine isomer showed higher thermal stability than the ones based on *meta*- and *para*-isomers [38]. In addition, as shown in Scheme 1, the norbornene functional group in BZ9 can play a terminal role of a main-chain type benzoxazine oligomer. The obtained main chain benzoxazine showed a relatively lower Mn (3036) and PDI (1.02), suggesting the *o*-norbornene functional phenol as an effective terminal functionality to control the molecular weights and narrow polydispersity. Besides, BZ9 exhibited higher thermal stability than the oligomer without the norbornene group as the glass transition temperature and char yield were as high as 360 °C and 66%, respectively [32].

## 3. Synthesis of Side-Chain Type Benzoxazine Resins via Ring-Opening Metathesis Polymerization (ROMP)

**Scheme 2:**
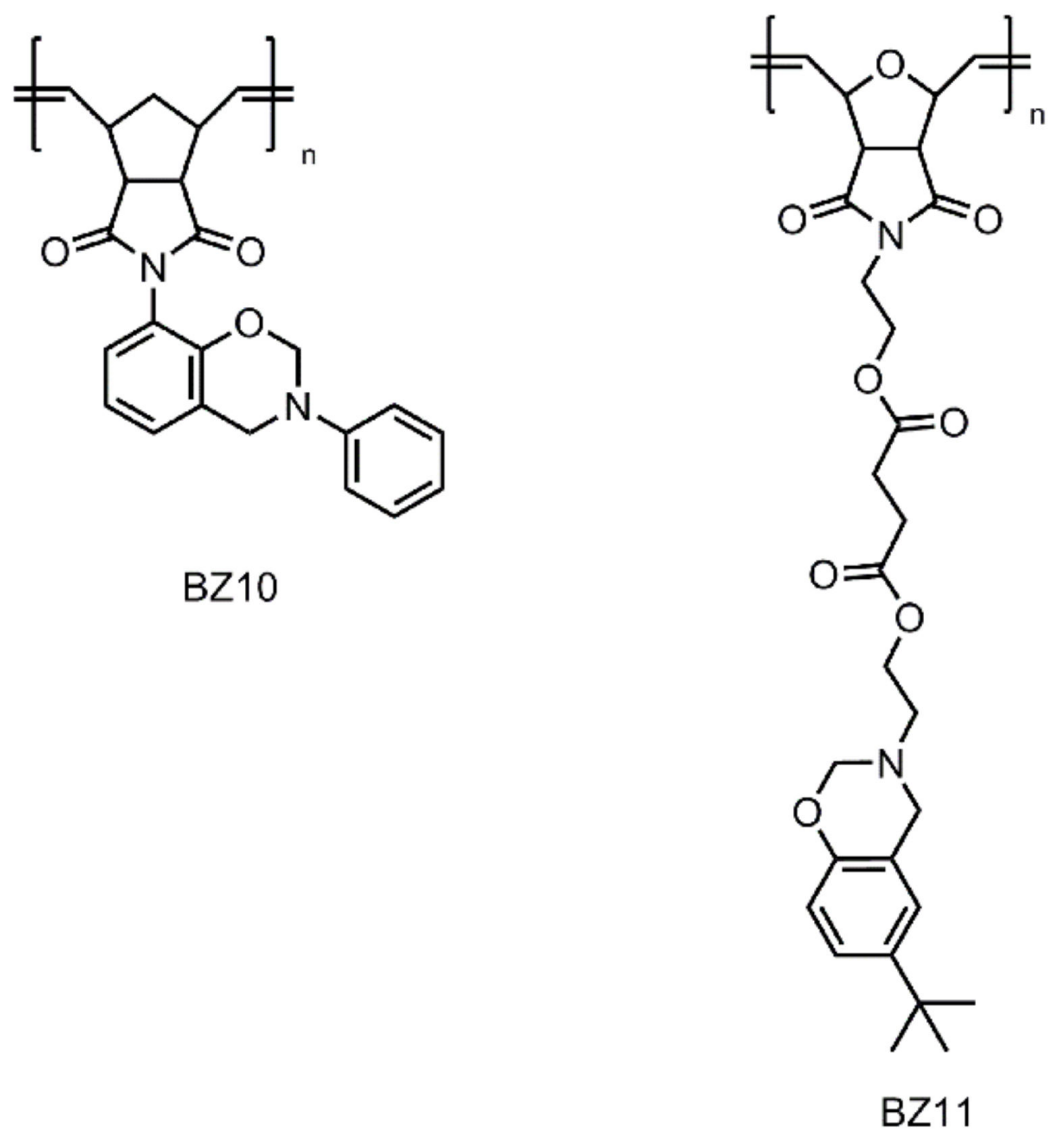
Norbornene-containing precursor via ROMP.

Main-chain type benzoxazine is a solution to overcome its original disadvantages: brittleness, difficulty of processing into a thin film from the typical monomers, and necessity of moderately high temperature for the ring-opening polymerization. Notably, ring-opening metathesis polymerization (ROMP) is an effective method for the chain-growth polymerization of norbornene using metal alkylidene initiators. The initiators for ROMP are generally applied by molybdenum and ruthenium complex catalysis. However, main-chain type benzoxazine based on ROMP has been seldomly researched, only two cases (BZ10 and BZ11) have been reported (Scheme 2) [39,40].

BZ10 was directly synthesized from BZ1 via ROMP; DSC analysis of BZ1 and BZ10 both showed a exothermic peak associated with curing from 280 °C to 340 °C with a peak maximum of 241 °C, indicating that ROMP does not evidently influence the ring-opening polymerization of benzoxazine. Interestingly, BZ10 possesses lower polymerization enthalpy, but higher activation energy than BZ1(BZ10: −△*H* = 120 J g^−1^, *E*_a_ = 148.8 kJ mol^−1^; BZ1: −△*H* = 173 J g^−1^, *E*_a_ = 90.4 kJ mol^−1^), because the high molecular weight and the rigidity originating from benzoxazine groups as the side chain decrease the mobility of the polymer chains of BZ10. The additional ROMP approach helps to increase the molecular weight of the thermoset precursor and can also prevent the decomposition of defects generated in the initial stages of the thermal decomposition of polybenzoxazines based on mono-functional benzoxazines. Thus, the thermal properties of BZ10-based polybenzoxazine are much better than the polybenzoxazine derived from BZ1. Tasdelen and Durmaz synthesized BZ11 through ROMP and the esterification reaction. The group focused on the relationship between molecular weight and property. As a result, the molecular weight of the polymers had no significant influence neither on the curing onset and maximum temperatures, nor on the glass transition temperature, decomposition temperature, and the char yield. The data for the thermal properties of polybenzoxazines derived from BZ1, BZ10, and BZ11 are summarized in Table 1.

Remarkably, even though the molecular weight of the synthesized BZ10 was not so high (BZ10: the number-average molecular weight and weight-average molecular weight were estimated to be 3840 and 7290 with a polydispersity index equaling 1.90; BZ11: The maximum number-average molecular weight and weight-average molecular weight were estimated to be 21,100 and 34,800 with a polydispersity index equaling 1.65), it should be emphasized that the molecular weight of both PBZ10 and PBZ11 are big enough for normal film-forming processing.

## 4. Development of High Performance Thermosets through the Hydrosilylation Reaction

**Scheme 3:**
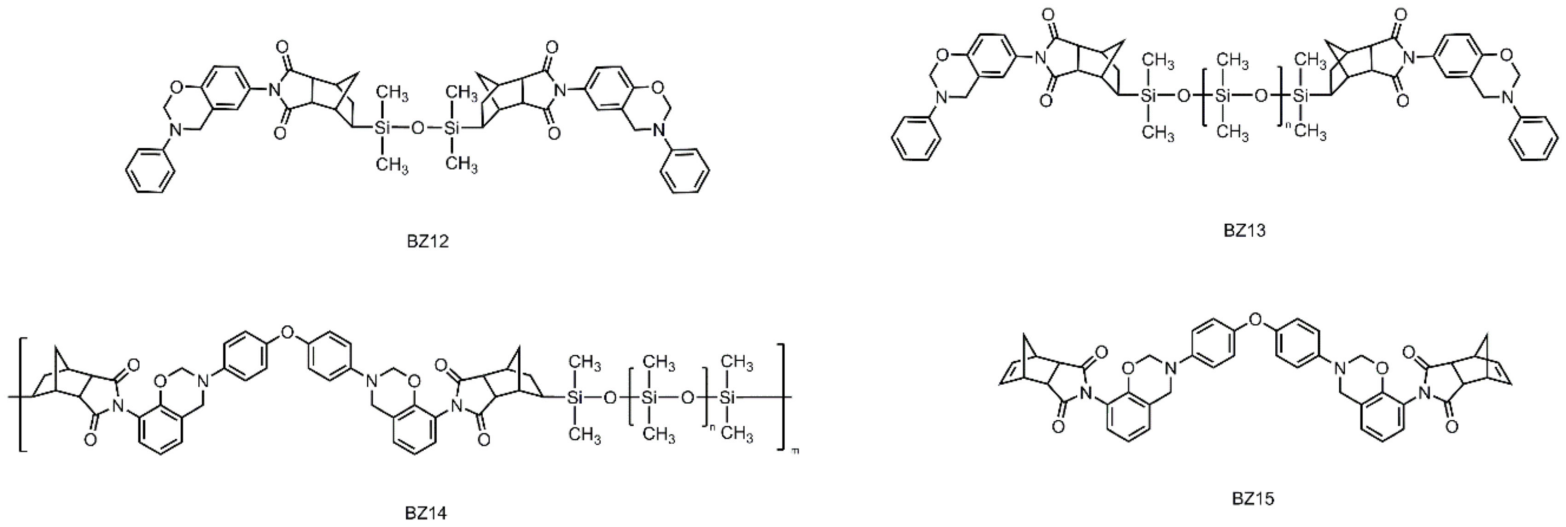
Chemical structures of BZ12–BZ15.

**Scheme 4:**
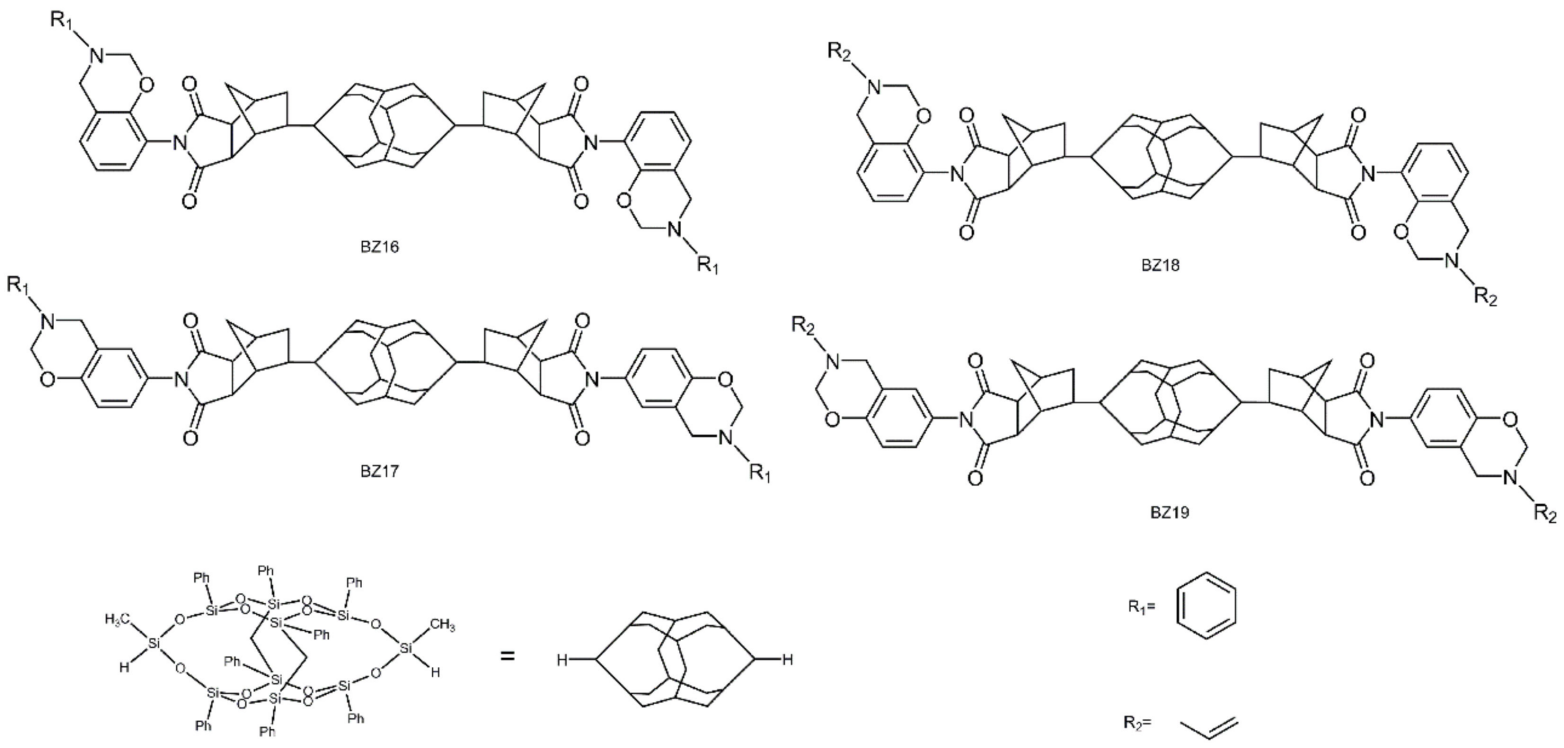
Chemical structures of BZ16–BZ19.

The special structure and composition of siloxane molecules make it both inorganic and organic, so it is believed to have the dual advantages of both organic and inorganic materials such as excellent thermal stability, low surface tension, good flexibility, low glass transition temperature, high air permeability, and excellent dielectric properties. As early as 2008, Hosta Ardhyananta, Mohd. Haniff Wahid, Masahiro Sasaki et al. prepared a polybenzoxazine-*co*-polydimethylsiloxane (PBZ–PDMS) hybrids by the blending method and sol–gel method, which have high mechanical properties and thermal properties [41]. The hydrosilylation reaction is an applicable method to combine siloxane and benzoxazines in one monomer, in addition, it makes the norbornene structure an ideal carrier to conduct the reaction because of the double bond in the structure. The idea might be a second approach to overcome the disadvantage mentioned in the former section. The chemical structures of siloxane-containing benzoxazines are shown in Scheme 3 and Scheme 4.

The pioneer work reported by Kai-Chi Chen, Hsun-Tien Li, Shu-Chen Huang et al. described the preparation of the benzoxazine BZ12 using paraformaldehyde, aniline, and the siloxane-containing dihydroxyl compound, which was synthesized through the hydrosilylation reaction [42]. Interestingly, BZ12 only contains a short segment of siloxane in its monomer, but reflects a huge improvement in thermal properties and surface behavior compared to the traditional benzoxazine monomer BA-a (bisphenol A and aniline derived benzoxazine monomer). Furthermore, the group designed and incorporated a longer siloxane segment into benzoxazines by altering tetramethyl disiloxane into polydimethylsiloxane (BZ13) [43]. The thermal properties of polybenzoxazines based on BZ12 and BZ13 are shown in Table 2. After curing in different conditions, all BZs revealed the same pattern: better thermal properties were exhibited when curing for a longer time. For another, when longer segments of siloxane were incorporated into structure, better thermal properties were shown. The surface properties of polybenzoxazines based on BZ12 and BZ13 are shown in Table 3. When longer siloxane segments were incorporated, the surface free energies decreased. However, surface free energy decreased more after a longer curing time due to the exposure of phenolic groups. As a result, the incorporation of siloxane segments improved the thermal stability while maintaining low surface free energy after thermal annealing at high temperatures for lengthy periods of time. Notably, DMA thermograms of the BZ13-based thermoset showed the presence of siloxane and imide moieties significantly improved the flexibility and toughness of PBZs without sacrificing their high *T*_g_ (*T*_g_ of PBZ13 is 184 °C), the storage modulus of BZ13-based thermoset at room temperature was 600–800 MPa, which was much lower than conventional *ortho*-norbornene functional polybenzoxazines, which makes BZ13 readily form a free-standing, bendable film.

Zhang et al. prepared a main chain-type poly(benzoxazine-*co*-imide-*co*-siloxane) (BZ14) by a facile approach [44]. The group synthesized the bisbenzoxazine monomer (BZ15) with *ortho*-norbornene using 4,4′-oxydianiline, paraformaldehyde and the *ortho*-norbornene phenol, then combined the monomer and PDMS through the hydrosilylation reaction. DSC analysis showed that the exotherm maximum for the ring-opening polymerization of BZ14 (248 °C) was much lower than BZ15 (316 °C), due to which the ring-opened structures formed from BZ14 acted as efficient initiators for the ring-opening polymerization of the oxazine rings. Moreover, BZ14 required a lower temperature to complete polymerization than BZ15 due to the absence of vinylene groups in BZ13. Both polybenzoxazines based on BZ13 and BZ14 exhibited good thermal stability. Interestingly, BZ15-based polybenzoxazine possessed higher thermal stability under N_2_ than under air, presumably because of the presence of less thermally stable segments of PDMS in BZ14. Chen and Kuo synthesized double-decker silsesquioxane (DDSQ)-functionalized benzoxazine BZ16-BZ19 [34]. The group focused their attention on the effect on *ortho*-imide and allyl groups because the DDSQ cage structures enhanced the thermal resistance after thermal curing. The thermal stability and char yield of polybenxoazines derived from above benzoxazines followed the order: BZ17 = BZ19 > BZ16 > BZ18, indicating that the presence of the aniline or allylamine functionality did not affect the thermal stability after thermal curing and the *ortho*-substitution of the aminophenol units in BZ16 and BZ18 led to lower thermal stability than the *para*-substitution of the aminophenol units. The data of the thermal properties for the polybenzoxazines derived from BZ14–BZ19 are shown in Table 4.

In comparison with classical systems, siloxane-containing benzoxazines both showed great thermal properties, and most importantly, they have become tougher and more flexible according to the DMA thermograms (the initial storage modulus of PBZ13 and PBZ14 were 729.5 and ca. 800 MPa, respectively [44,45], while the initial storage modulus of both PBZ3 and PBZ5 were over 1500 MPa [31,38]). As a result, it was much easier to obtain free-standing films without adding plasticizers, since the PBZs exhibited superior flexibility and toughness [43].

## 5. Concluding Remarks and Future Outlook

As a thermosetting resin with application potential, the development of polybenzoxazine has focused on how to overcome the high complete thermal curing temperature and brittleness of the cured material. The design flexibility of the benzoxazine molecule provides an important means to overcome its shortcomings. Among the various functionalities, the introduction of norbornene functional groups effectively solves these problems, and its effect is manifested in a variety of ways. First of all, norbornene can polymerize and participate in the ring-opening polymerization of the benzoxazine ring under thermal initiation, which can increase the degree of polymerization of benzoxazine, reduce its curing temperature, and improve thermal stability. Second, norbornene has a ring opening metathesis polymerization effect, and can polymerize in the presence of a suitable catalyst when it exists in a benzoxazine molecule to obtain a linear molecule with an oxazine functional group on the side chain, which can undergo ring-opening polymerization to obtain high-molecular-weight polymers under thermal conditions. In addition, the presence of carbon–carbon double bonds in norbornene allows it to participate in the hydrosilylation reaction, so the obtained benzoxazine resin has good thermal stability and flexibility. Due to the features above-mentioned, norbornene functional benzoxazine resins have great potential to be applied at a high temperature and high pressure environment compared to traditional thermosetting resins, in addition, carbon–carbon double bonds in norbornene and various amine sources allow for further molecular design, which means that the system can be applied in different areas. However, the currently obtained high-performance norbornene based benzoxazine resin material still has a certain distance from its actual commercial application. It is significant to further solve the existing defects under the condition of maintaining the existing excellent performance and reduce the production costs. Blending with existing high-performance materials such as epoxy resin may be an effective means to reduce costs and expand production. Meanwhile, resins with high nitrogen content or silicone-based resins and some other resins with outstanding properties may also be efficiently added in the polymer system. At the same time, exploiting the diversity of norbornene group reactions in the benzoxazine field and the flexible molecular design of benzoxazine itself to further develop thermosetting resins with higher performance is also a promising direction for the research and application of norbornene-functionalized benzoxazines in the future.

## Figures and Tables

**Table 1:** Thermal properties of polybenzoxazines derived from BZ1, BZ10, and BZ11 [39,40].

Monomer	T_5_ (°C)	T_10_ (°C)	Char Yield (wt%)
BZ1	319	366	39 (850 °C)
BZ10	371	431	52 (850 °C)
BZ11	ca. 360	ca. 395	ca. 43 (800 °C)

**Table 2:** The thermal properties o polybenzoxazines derived from BA-a, BZ12, and BZ13 [43].

Monomer	Curing Time (h)		T_5_ (℃)	T_10_ (°C)	T_d_ (°C)	Char Yield (wt%)
	at 200 °C	at 230 °C				
BA-a	2	2	334.6	356.8	334.2	34.3
		4	328.8	360.7	342.7	42.7
		6	336.5	369.8	341.6	46.3
BZ12	2	2	355.7	417.8	452.9	48.0
		4	361.5	427.2	448.4	48.4
		6	358.5	415.8	446.5	49.3
BZ13	2	2	437.1	474.2	471.0	45.1
		4	437.2	481.3	459.9	48.1
		6	430.6	477.4	463.7	50.9

**Table 3:** The surface properties of polybenzoxazines derived from BA-a, BZ12, and BZ13 [43].

Monomer	Curing Conditions		Contact Angle (°)			Surface Free Energy, γ_s_ (mJ m^−2^)
	Temperature	Time (h)	Water	EG	DIM	
BA-a	230	1	106.1	82.5	84.3	16.6
		2	93.9	70.0	76.3	22.5
		4	78.8	55.6	68.6	30.0
		8	59.4	25.5	53.8	43.6
		24	13.7	20.3	45.8	42.6
BZ12	230	1	108.8	85.4	86.8	15.1
		2	108.0	83.5	83.6	16.6
		4	106.5	82.5	83.1	17.0
		8	99.4	74.7	78.2	20.4
		24	84.0	69.3	71.5	24.6
BZ13	230	1	113.7	93.9	92.3	12.4
		2	112.2	89.0	86.9	14.7
		4	111.9	88.7	86.8	14.7
		8	110.9	88.2	86.8	14.8
		24	108.3	82.3	84.3	16.2

**Table 4:** Thermal properties of polybenzoxazines derived from BZ14–BZ19 [44,45].

Polymer	Atmosphere	T_5_ (°C)	T_10_ (°C)	Char Yield (wt%)
PBZ14	N_2_	429	461	46.0 (850 °C)
	air	400	436	45.0 (850 °C)
PBZ15	N_2_	400	431	55.0 (850 °C)
	air	412	449	15.0 (850 °C)
PBZ16	N_2_	/	494	72.7 (800 °C)
PBZ17	N_2_	/	512	75.0 (800 °C)
PBZ18	N_2_	/	471	67.2 (800 °C)
PBZ19	N_2_	/	512	75.0 (800 ℃)
